# Jak2/STAT6/c-Myc pathway is vital to the pathogenicity of Philadelphia-positive acute lymphoblastic leukemia caused by P190^BCR-ABL^

**DOI:** 10.1186/s12964-023-01039-x

**Published:** 2023-01-31

**Authors:** Run Qin, Teng Wang, Wei He, Wei Wei, Suotian Liu, Miao Gao, Zhenglan Huang

**Affiliations:** 1 Key Laboratory of Laboratory Medical Diagnostics Designated By the Ministry of Education, Department of Clinical Hematology, School of Laboratory Medicine, Chongqing Medical University, Chongqing, China; 2 Department of Hematology, The Second Affiliated Hospital of Chongqing Medical University, Chongqing, China; 3 Department of Laboratory Medicine, The First Affiliated Hospital of Chongqing Medical University, No. 1, Youyi Road, Yuzhong District, Chongqing, 400016 China

**Keywords:** Philadelphia-positive chronic myeloid leukemia, Philadelphia-positive acute lymphoblastic leukemia, STAT6, c-Myc, Jak2

## Abstract

**Background:**

The Philadelphia chromosome encodes the BCR-ABL fusion protein, which has two primary subtypes, P210 and P190. P210 and P190 cause Philadelphia-positive chronic myeloid leukemia (Ph+ CML) and Philadelphia-positive acute lymphoblastic leukemia (Ph+ ALL), respectively. The Ph+ ALL is more malignant than Ph+ CML in disease phenotype and progression. This implies the key pathogenic molecules and regulatory mechanisms caused by BCR-ABL in two types of leukemia are different. It is reported that STAT6 was significantly activated only in P190 transformed cells. However, the potential role and the mechanism of STAT6 activation in Ph+ ALL and its activation mechanism by P190 are still unknown.

**Methods:**

The protein and mRNA levels of STAT6, c-Myc, and other molecules were measured by western blot and quantitative real-time PCR. The STAT6 inhibitor AS1517499 was used to specifically inhibit p-STAT6. The effect of p-STAT6 inhibition on Ph+ CML and Ph+ ALL cells was identified by CCK-8 and FCM assay. Dual luciferase reporter and ChIP assay were performed to confirm the direct binding between STAT6 and c-Myc. The impact of STAT6 inhibition on tumor progression was detected in Ph+ CML and Ph+ ALL mouse models.

**Results:**

Our results demonstrated that P210 induced CML-like disease, and P190 caused the more malignant ALL-like disease in mouse models. STAT6 was activated in P190 cell lines but not in P210 cell lines. Inhibition of STAT6 suppressed the malignancy of Ph+ ALL in vitro and in vivo, whereas it had little effect on Ph+ CML. We confirmed that p-STAT6 regulated the transcription of c-Myc, and STAT6 was phosphorylated by p-Jak2 in P190 cell lines, which accounted for the discrepant expression of p-STAT6 in P190 and P210 cell lines. STAT6 inhibition synergized with imatinib in Ph+ ALL cells.

**Conclusions:**

Our study suggests that STAT6 activation plays an essential role in the development of Ph+ ALL and may be a potential therapeutic target in Ph+ ALL.

**Video abstract**

**Supplementary Information:**

The online version contains supplementary material available at 10.1186/s12964-023-01039-x

## Background

BCR-ABL is an oncoprotein with constitutive tyrosine kinase, which is encoded by the BCR-ABL fusion gene located in the Philadelphia (Ph) chromosome. The BCR-ABL fusion gene is produced when the breakpoint cluster region (BCR) gene fuses to the Abelson tyrosine kinase (ABL) gene due to a t (9;22) reciprocal translation [1]. Because of the different translocation breakpoints in the BCR gene, different BCR-ABL fusion protein subtypes are produced. P210 and P190 are the most common BCR-ABL isoforms [2–4]. Except for the absence of Dbl-homology (DH) domain and Pleckstrin-homology (PH) domain in P190, the other sequences and protein domains are identical of both two isoforms [5].

However, P190 and P210 lead to two different kinds of leukemia – Philadelphia-positive acute lymphoblastic leukemia (Ph+ ALL) and Philadelphia-positive chronic myeloid leukemia (Ph+ CML). Ph+ CML caused by P210 is typically characterized by excessive proliferation of mature myeloid cells with normal differentiation and sluggish disease progression [2, 6]. The treatment and prognosis of Ph+ CML patients have substantially improved as a result of the successful application of tyrosine kinase inhibitors (TKIs), such as the first-generation TKI imatinib (IM). Additionally, the second-generation TKIs as dasatinib and nilotinib are developed for patients with IM resistance [7, 8]. Anyway, these TKIs target the BCR-ABL kinase region and bring great success in clinical practice with a 5-year survival rate up to 90% [9, 10]. While P190 occurs in most Ph+ ALL patients, which is characterized by complete retarded differentiation of lymphocytes and a rapid onset and progression [11, 12]. Haematopoietic stem cell transplantation (HSCT) has been the gold standard therapy for patients with Ph+ ALL. The introduction of targeted therapy using TKIs has revolutionized the therapy [13]. However, since P190 is associated with an increased risk of acute progression and is prone to mutation, TKIs have poor efficacy in patients with Ph+ ALL, and the overall survival rate is still low [14, 15]. In conclusion, different BCR-ABL isoforms cause different leukemic diseases with significant biological differences. This implies the key pathogenic molecules and regulatory mechanisms caused by BCR-ABL in two types of leukemia are different. Hence, to explore these differences in the development of two subtypes of BCR-ABL positive leukemia may provide meaningful value for further understanding BCR-ABL-related pathogenesis and improving the treatment effect of leukemia.

Jak/STAT signaling is a critical BCR-ABL downstream pathway, which plays a vital role in modulating the survival, proliferation, and differentiation of leukemia cells [16–18]. Convincing evidence has proved that abnormal activation of STAT3 and STAT5 plays a critical role in the occurrence and development of Ph+ CML and Ph+ ALL [19–21]. That means STATs play an important role in the pathogenicity of Ph-positive leukemia. Moreover, it is reported that the phosphorylation levels of STATs activated by BCR-ABL is different between BCR-ABL subtypes [22–24]. Ilaria R L et al. [22] showed that STAT1, STAT3, and STAT5 were constitutively activated in both P210 and P190 transformed cells, but STAT6 was significantly activated only in P190 transformed cells. However, the meaning of the different activation of STAT6 in Ph+ ALL caused by P190 is still unknown. If there is a relationship between the difference of STAT6 activation and the significant biological differences in Ph+ leukemia is worth to be investigated. To explore the related mechanism of STAT6 activation in P190 cells contributes to illustrate the mechanism of more serious disease characteristics and improve the treatment effect of Ph+ ALL caused by P190.

In this study, we identified that P210 induced CML-like disease, whereas P190 caused the more malignant ALL-like disease in mouse models, and STAT6 was activated in P190 cell lines but not in P210 cell lines. Inhibition of STAT6 suppressed Ph+ ALL progression in vitro and in vivo but had little effect on Ph+ CML cells. Furthermore, we demonstrated that STAT6 was activated by Jak2 and promoted Ph+ ALL progression by regulating c-Myc transcription. Notably, STAT6 inhibition had a synergistic effect with IM in Ph+ ALL cells. Thus, our results suggest that Jak2/STAT6/c-Myc pathway contributes to the progression of Ph+ ALL, and inhibition of this pathway may provide a new potential therapy for Ph+ ALL patients.

## Methods

### Reagents

The inhibitors, such as AS1517499, Fedratinib, and IM were purchased from Targetmol (USA) and stored at – 20 °C after being dissolved in DMSO.

### Cell lines and cell culture

SUP-B15 is a human Philadelphia chromosome positive acute lymphocytic leukemia cell line and was donated by doctor Wang Teng from the Second Affiliated Hospital of Chongqing Medical University. K562 is a human Philadelphia chromosome positive chronic myeloid leukemia cell line and was purchased from the Cell Culture Center of the Chinese Medical Science Academy in Shanghai. BP210 is a murine Philadelphia chromosome positive chronic myeloid leukemia cell line which is constructed by stably transferring BCR-ABL-expressing retroviral vector MIGR1-P210 (P210^BCR-ABL^) into Ba/F3 cells followed by selection with limiting dilution analysis [25, 26]. P210^BCR-ABL^ was kindly proved by Dr. Warren S. Pear. BP190 is a murine Philadelphia chromosome positive acute lymphocytic leukemia cell line and was donated by Professor O Hantschel [27]. Ba/F3 was cultured in the RPMI 1640 medium, including 10% fetal bovine serum (FBS) and 10 ng/ml murine IL-3 (R&D, USA). BP210, BP190, and K562 cell lines were cultured in RPMI 1640 medium with 10% FBS. SUP-B15 cell line was cultured in the Iscove’s Modified Dulbecco’s Medium (IMDM) supplemented with 20% FBS. All these cells were maintained in an incubator at 37 °C with 5% CO_2_.

### Mouse models

Six- to Seven-week-old female BALB/C mice were injected with 5 × 10^6^ BP210 or BP190 cells suspended in 200 μL PBS via the tail vein. After one week, the mice injected with BP210 or BP190 cells were divided into two groups at random (n = 8 for each group): one group was injected with AS1517499 (20 mg/kg) every other day for five consecutive times by intraperitoneal administration, the other group was treated with PBS by the same manner. The mice’s body weight and the white blood cells (WBCs) count of peripheral blood were measured weekly. Wright’s staining and hematoxylin and eosin (HE) staining of the heart, liver, spleen, lung, kidney, and bone marrow (BM) were used to assess the infiltration of leukemic cells. The Biomedical Ethics Committee approved all animal experiments at Chongqing Medical University (2,022,103).

### Reverse transcription PCR and quantitative real-time PCR (qRT-PCR)

Total RNA samples were extracted with the TRIZOL reagent (Takara, Japan). The reverse transcription of total RNA is performed by using the prime Script RT reagent Kits (Takara, Japan). Quantitative real-time PCR was performed by using the TB Green PCR Kit (Takara, Japan). Human β-actin was used as an internal reference for human cell lines (K562, SUP-B15), and mouse GAPDH was used for mouse-derived cell lines (Ba/F3, BP210, and BP190). Primer sequences are provided in Table 1.

**Table 1:** The list of primer sequences used for the qPT-PCR

Gene	Forward primer (5′-3′)	Reverse primer (3′-5′)
Actin(human)	ACTTAGTTGCGTTACACCCTT	TGTCACCTTCACCGTTCC
STAT6(human)	GTTTACAGTGAAGAAGGCCCG	CTGGGCTGGCCCTAAAAACT
c-Myc(human)	GGCTCCTGGCAAAAGGTCA	CTGCGTAGTTGTGCTGATGT
Jak2(human)	CGAATGGTGTTTCTGATGTACC	GTCTCCTACTTCTCTTCGTACG
GAPDH(mouse)	CAAGGTCATCCTGACAACTTTG	GTCCACCACCCTGTTGCTGTAG
STAT6(mouse)	ATCTTCAACGACAACAGCCTCA	GGAGAAGGCTAGTGACATATTG
c-Myc(mouse)	GGACAGTGTTCTCTGCC	CGTCGCAGATGAAATAGG
Jak2(mouse)	CTTCCACATAGACGAGTCAACCA	GTTCTGCTGCTGCCACTACA

### Western blot assay

The western blot assay was performed to detect the protein expression. The total proteins were obtained by RIPA buffer (CST, USA), and the concentration was measured by BCA protein assay (Beyotime, China). An equal amount of proteins (80 μg) were electrophoresed through sodium dodecyl sulfate–polyacrylamide gel (SDS-PAGE). Then protein strips were transferred to the PVDF membranes (Millipore, USA). The membrane was blocked with 5% nonfat milk. And then incubated with the corresponding primary antibody overnight, including anti-STAT6 (Abcam, USA), anti-p-STAT6 (Abcam, USA), anti-p-BCR-ABL (CST, USA), anti-Caspase-3 (CST, USA), anti-PARP (CST, USA), anti-p27 (CST, USA), anti-p21 (Bimake, USA), anti-Cdk1/Cdc2 (Bimake, USA), anti-Cdc25c (Bimake, USA), anti-PCNA (Bimake, USA), anti-c-Myc (CST, USA), anti-Jak1 (CST, USA), anti-Jak2 (CST, USA), anti-p-Jak2 (CST, USA), anti-Jak3 (CST, USA), anti-Actin (Sangon Biotech, China). Then the membrane was incubated with the corresponding secondary antibody. The protein signal was developed with Chemistar™ high-sig ECL Western Blot Substrate (Tanon, China).

### Cell counting kit-8 (CCK-8) assay

The cell viability was evaluated by CCK-8 assay (TOP SCIENCE, China). 4 × 10^3^ cells were plated into each well in 96-well plates. Then cells were treated with different concentrations of AS1517499 for 48 h. At the specified time, CCK-8 reagent was added and incubated at 37 °C for 3 h. The optical density (OD) was measured at 450 nm (Eon, BioTek, USA). The drug concentration resulting in the 50% inhibitory concentration (IC_50_) was determined. The cell survival rate of each group treated with different concentrations of AS1517499 was obtained, and the IC_50_ was calculated with GraphPad Prism 8.0 software. For calculating the combination index (CI) value, the absorbance value of each well in the 96-well plate was measured in the CCK-8 assay. The cell survival rate of each group with different concentrations of AS1517499 and IM was calculated with the formula: survival rate = (ODtreatment-ODblank)/(ODcontrol-ODblank). Then the CI was calculated by the CompuSyn software. There were 3 duplicates for each group.

### Cell count assay

The cell proliferation was assessed with the cell count assay. The cells were collected and adjusted to the concentration of 4 × 10^3^ per well in 96-well plates. Then cells were treated with various concentrations of AS1517499 for 48 h. Cell number in each group was counted. Each group had 3 duplicate wells.

### Colony formation assay

The colony formation assay was used to assess the cell self-renewal ability. The cells were collected and seeded in the semisolid culture-medium in 96-well plates (100 cells/well). After 7 days, the number of colonies was counted and the pattern of colonies was captured with an inverted microscope. Each group had three repeats.

### Cell cycle and apoptosis analysis

Cells (8 × 10^5^) were planted in 6-well plates and treated with identified concentrations of AS1517499 for 48 h. After that, each cell was collected and washed 3 times with PBS. For cell cycle analysis, harvested cells were fixed in ice-cold 70% ethanol at 4 °C overnight. Then cells were washed with PBS, re-suspended with 500 μL PI/RNase solution, and incubated in darkness for 0.5 h before detection by flow cytometry (FCM). To analyze the cell apoptosis, the harvested cells were double-labeled with annexin V and PI, then measured by FCM according to the manufacturer’s instructions.

### Immunophenotyping analysis

8 × 10^5^ cells were plated to each well in a 6-well plate. Each well was added with various concentrations of AS1517499 and incubated in a cell incubator. After 48 h, cells in each group were collected and stained with fluorescein labeled antibodies. Then the immunophenotyping was analyzed by FCM. This assay was repeated 3 times.

### Immunofluorescence (IF) assay

Cells were collected, washed, and smeared on the slides. The cells were fixed with 4% paraformaldehyde, permeabilized with 1% Triton X-100, and blocked with goat serum. After that, the cells were incubated with corresponding primary antibodies, including anti-c-abl (Santa USA), anti-STAT6 (Abcam, USA), anti-p-STAT6 (CST, USA), and anti-c-Myc (Abmart, China), at 4 °C overnight. The next day, the cells were incubated with Cy-3 or FITC labeled secondary antibody (Invitrogen, USA). Then, the nucleus was stained with 4.6-diamidino-2-phenylindole (DAPI). Finally, the slides were sealed with glycerin and observed with the confocal microscope.

### Dual luciferase reporter assay

The dual luciferase reporter assay was used to explore the direct binding between STAT6 and c-Myc. The sequence of STAT6 was cloned into the pcDNA3.1 vector. The potential promoter region of c-Myc were divided into two overlapping segments, which named P1 and P2 respectively, then cloned them into the pGL3 plasmid. Subsequently, 293T cells were co-transfected with either P1 or P2 along with STAT6 using Lipo8000 (Beyotime, China). The pcDNA3.1 empty vector was served as control. After 48 h, luciferase activities were tested with the Dual Luciferase Reporter Assay System (Promega, USA). Renilla luciferase activity was normalized to firefly luciferase activity.

### Chromatin immunoprecipitation (ChIP)

The ChIP assay was carried out according to the instructions of the ChIP assay kit (CST, USA). In brief, cells (1 × 10^8^) were collected and treated with 1% formaldehyde to generate DNA–protein cross-links. Next, centrifugation and the residue were sonicated. Then the solution was treated with magnetic beads. Then the lysates were incubated with p-STAT6 antibody (CST, USA) or IgG antibody overnight. Finally, the DNA fragments were quantified by qPCR with the specific primers. The sequences are provided in Table 2.

**Table 2:** The list of primer sequences used for the ChIP-qPCR

Gene fragment sequence	Forward primer (5′-3′)	Reverse primer (3′-5′)
1	AGGCTGAGGTAGGAGGT	GCAGAAGGTGATGGGTA
2	TTCTGCTGTGCCTCCC	GTAATGGCAAACGTGAAATA
3	GGGTGATGTTCATTAGCAG	GTCCGAAGAAAGAGGAGTTA
4	GAATAACAAGGAGGTGGCT	GGTTTGCGAAAGTAAAGTAAG

### Statistical methods

Statistical analysis was performed by GraphPad Prism 8.0 software. All data were presented as a mean ± SD. Student’s t-test or one-way ANOVA was used to assess statistical difference between groups. The statistical significance was assigned when *P* values were < 0.05(*).

## Results

### P190 induces more aggressive leukemia than P210 in mice

**Fig. 1:**
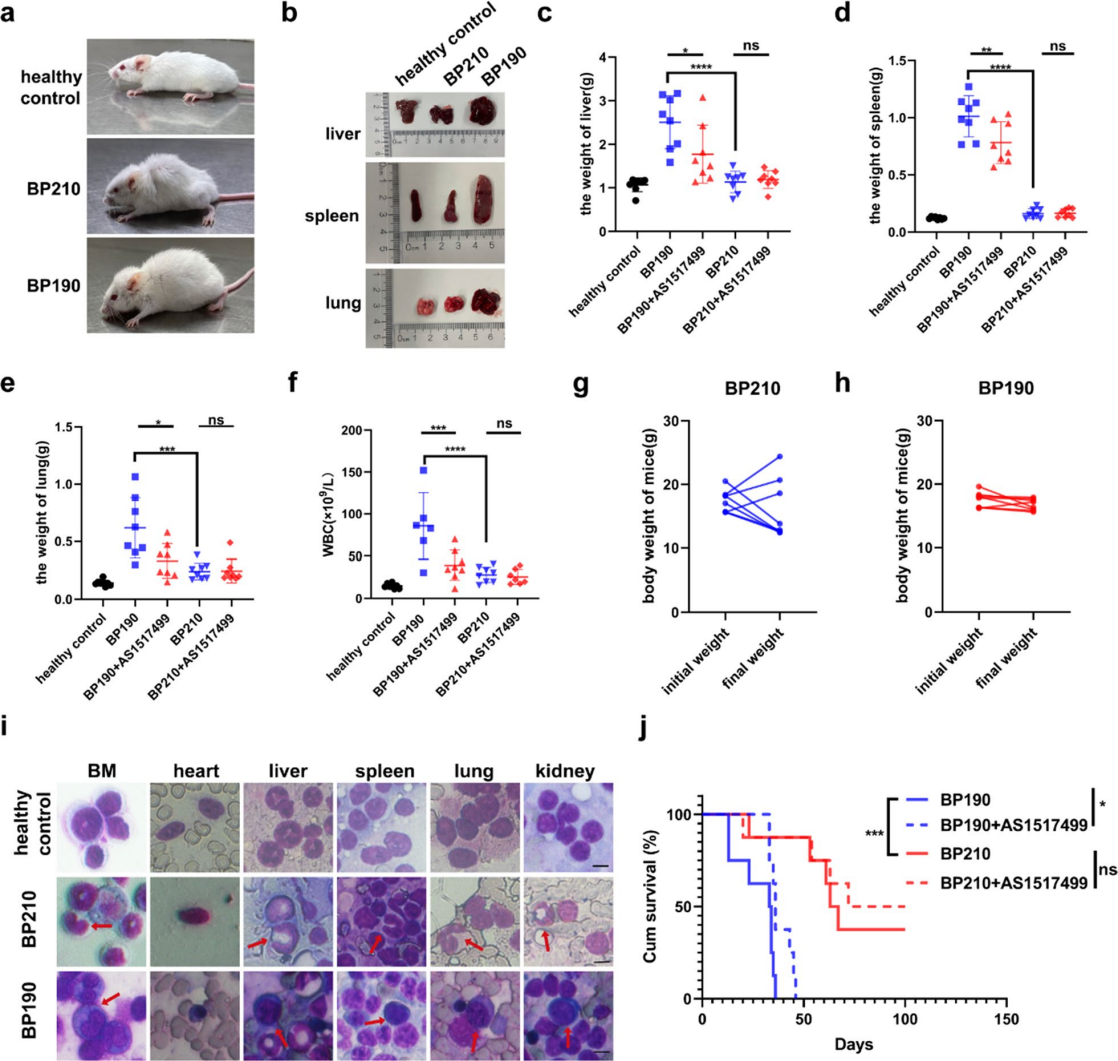
P190 induces more aggressive leukemia than P210 in mice. **a** Representative diseased mice in P210 and P190 groups were photographed for comparison. **b** Representative liver, spleen, and lung in groups of healthy control, BP210, and BP190 were photographed for comparison. The weights of (**c**) liver, (**d**) spleen, and (**e**) lung were measured and analyzed. **f** The maximum WBC counts of mice were determined and analyzed (The dynamic WBC counts were absent because two mice in the P190 control group and one in the P210 treated group died prematurely). The mice’s weight changes from the beginning to the end in the (**g**) P210 and (**h**) P190 group were graphed. **i** The infiltration of leukemic cells in the heart, liver, spleen, lung, kidney, and BM was analyzed by Wright’s staining, the stab or segmented granulocytes cells in P210 group and immature lymphocytes in P190 group were indicated by red arrows, scale bar, 5 μM. **j** Kaplan–Meier survival analysis of mice. ***p < 0.001, ****p < 0.0001. ns indicates no significant differences

To compare the pathogenicity of P210 and P190, we constructed two mouse leukemia models with the same number of BP210 and BP190 cells (Additional file 1: Fig. S1a). Firstly, we observed that the mice’s conditions in the two models varied significantly at the beginning. The diseased mice in the P210 group displayed arched back, whereas the diseased in the P190 group had chest and abdomen swelling and hemorrhagic malignant pleural effusion (Fig. 1a). We also examined the alterations in some visceral organs. As shown in Fig. 1b, the hepatosplenomegaly and pulmonary hemorrhage of mice in the P190 group were more severe than those in the P210 group. We also found that the weights of mouse liver, spleen, and lung in the P190 group were heavier than those in the P210 group (Fig. 1c–e). However, there was no discernible difference in the P210 group (Additional file 1: Fig. S1b–d). The WBCs were also significantly higher in the P190 group than in the P210 group (Fig. 1f). In addition, the analysis of mice’s weight changes from the beginning to the end showed that the weight of disease mice in the P210 group was significantly reduced. In contrast, the weight changes of disease mice in the P190 group were not insignificant (Fig. 1g, h). Then we analyzed the organ infiltration of leukemic cells with Wright’s staining. The BM, as well as liver, spleen, lung, and kidney, were infiltrated by rod-shaped or lobulated granulocytes in the P210 group. In contrast, immature lymphocytes heavily populated in the BM, heart, liver, spleen, lung, and kidney in the P190 group (Fig. 1i). Finally, mice in the P190 group had a relatively shorter disease incubation and shorter lifetime according to the survival analysis. Two mice developed leukemia as early as ten days, and the other six mice perished after 4–6 weeks. Whereas in the P210 group, one mouse developed disease approximately after three weeks, and four mice died between 8 and 12 weeks, and three mice were still alive at 100 days. The morbidity was 100% in P190 group and 70% in P210 group. The mean lifetime was 33.5 days in P190 group and 65 days in P210 group (Fig. 1j). In conclusion, these results showed that P190 induced more severe leukemia with a rapid onset and quick demise, as well as more serious infiltration than P210.

### STAT6 is activated by BCR-ABL in P190 cell lines, and P190 cell lines is more sensitive to the inhibition of STAT6 activation than P210 cell lines

**Fig. 2:**
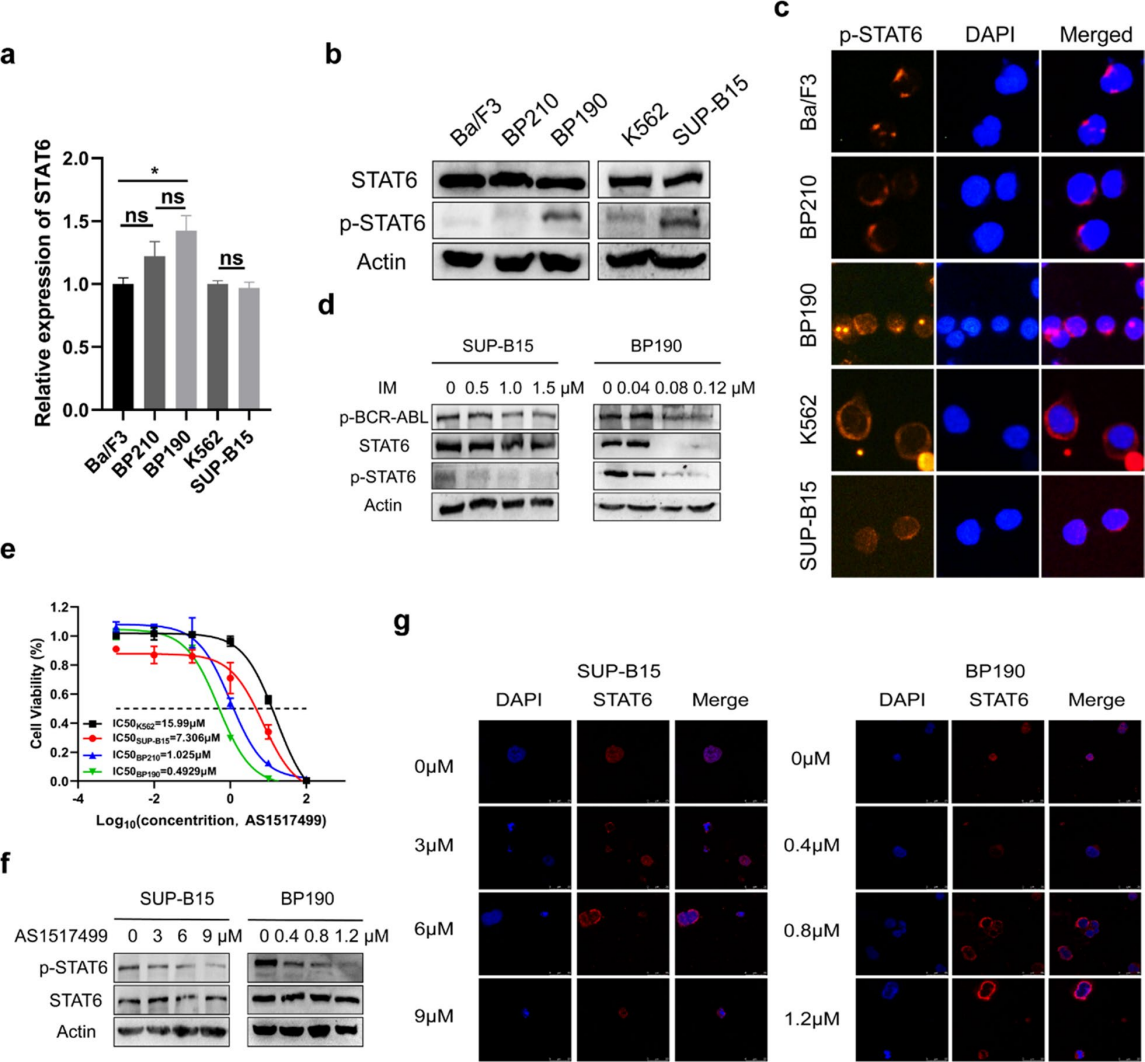
STAT6 is activated by BCR-ABL in P190 cell Lines, and P190 cell lines is more sensitive to the inhibition of STAT6 activation than P210 cell lines. **a** The mRNA levels of STAT6 in Ba/F3, BP210, BP190, K562, and SUP-B15 cells were analyzed by qRT-PCR assay. **b** The protein levels of STAT6 and p-STAT6 in Ba/F3, BP210, BP190, K562, and SUP-B15 cells were analyzed by western blot assay. **c** The expression of p-STAT6 was detected by IF assay. **d** The expression of BCR-ABL, STAT6, and p-STAT6 in BP190 and SUP-B15 cells treated with IM for 48 h was detected by western blot assay. **e** The IC_50_ values of AS1517499 treated BP210, BP190, K562 and SUP-B15 cells for 48 h were measured by CCK-8 assay. SUP-B15 and BP190 cells treated with different concentrations of AS1517499 for 48 h, **f** the expression of p-STAT6 and STAT6 was measured by western blot assay, **g** and the expression of STAT6 in nucleus and cytoplasm was measured by IF assay. *p < 0.05. ns indicates no significant differences

To verify whether there is a difference in the expression of STAT6 in P190 cells and P210 cells, we analyzed the mRNA, protein and phosphorylation level of STAT6 in Ba/F3 cells, P210 cell lines (BP210 and K562 cells), and P190 cell lines (BP190 and SUP-B15 cells) by qRT-PCR and western blot assays. The results showed p-STAT6 was significantly activated in P190 cell lines but not in P210 cell lines, although there was no difference in the protein and mRNA expression of STAT6 between P210 and P190 cell lines (Fig. 2a, b). Consistently, the fluorescence intensity indicated that the protein level of p-STAT6 was much higher in P190 cell lines than in P210 by IF assay (Fig. 2c). However, p-STAT6 signal appeared in the cytoplasm of Ba/F3, BP210 and K562 cells, which may be due to the poor specificity of p-STAT6 antibody. Then we wanted to explore whether the activation of STAT6 was regulated by BCR-ABL kinase. We first examined the 50% inhibitory concentration (IC_50_) values of IM in BP190 and SUP-B15 cells by CCK-8 assay, and then selected a specific concentration gradient for the subsequent experiment based on the IC_50_ value. The results of the western blot assay showed that p-STAT6 was decreased when p-BCR-ABL was inhibited by IM (Fig. 2d). This result indicating that STAT6 activation was regulated by BCR-ABL kinase.

There is a significant difference in the activation of STAT6 between P190 and P210 cells. Then we tried to explore the influence of p-STAT6 inhibition in these BCR-ABL-positive cells. AS1517499 which is a specific inhibitor for p-STAT6 was selected to inhibit p-STAT6 [28]. The results showed that the IC_50_ values were much lower in P190 cell lines than in P210 cell lines (Fig. 2e). That meant P190 cells were more sensitive to p-STAT6 inhibitor than P210 cells. Next, we selected specific drug concentration gradients according to the IC_50_ values for the following effect trails to better explore the role of STAT6 activation in the Ph+ ALL development. The result of western blot showed that the expression of p-STAT6 was markedly inhibited by AS1517499 in SUP-B15 and BP190 cells (Fig. 2f). STAT6 usually locates in the cytoplasm. It translocated to the nucleus when it is phosphorylated. Hence, we detected the location of STAT6 by IF assay. The result revealed that a substantial of STAT6 translocated from the nucleus to the cytoplasm after AS1517499 treatment (Fig. 2g). This result indicated that the phosphorylation of STAT6 was effectively inhibited by AS1517499. Collectively, we found that STAT6 was activated in P190 cell lines but not in P210, and its activation was regulated by BCR-ABL kinase. AS1517499 can effectively inhibit the activation of STAT6. Importantly, P190 cell lines are more sensitive to the inhibition of STAT6 activation than P210 cell lines.

### *Inhibition of p-STAT6 suppresses the proliferation in Ph*+ *ALL cells more obvious than in Ph*+ *CML cells*

**Fig. 3:**
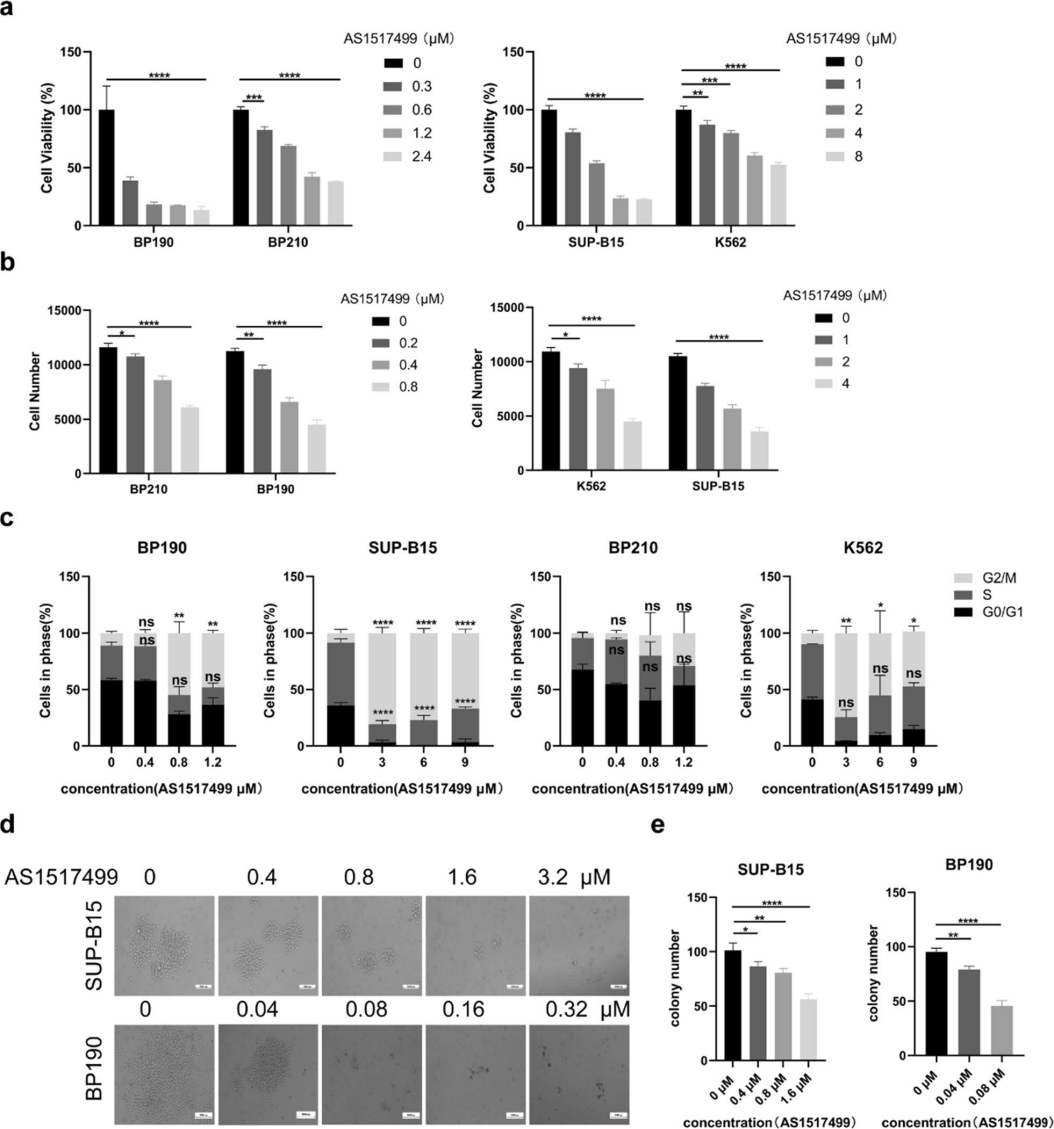
Inhibition of p-STAT6 suppresses the proliferation in Ph+ ALL cells more obvious than in Ph+ CML cells. Each indicated cell line treated with different concentrations of AS1517499 for 48 h, **a** cell viability was evaluated by CCK-8 assay; **b** cell proliferation was evaluated by cell counting assay; **c** cell cycle distribution was analyzed by FCM. **d** The self-renewal ability of BP190 and SUP-B15 cells treated with different concentrations of AS1517499 for seven days were evaluated by colony formation assay. **e** The colony numbers of three holes in each group were analyzed. *p < 0.05, **p < 0.01, ***p < 0.001, ****p < 0.0001. ns indicates no significant differences

We have proven that STAT6 is specifically activated in P190 cells. However, the significance of the activation of STAT6 in Ph+ ALL is still unknown. So we down-regulated p-STAT6 by its specific inhibitor AS1517499, then we detected the biological effect of p-STAT6 inhibition on the P190 cells. Firstly, we explored the impact of p-STAT6 on the proliferation of Ph+ ALL cells. We treated P210 and P190 cell lines with a series of concentrations of AS1517499 for 48 h, then detected cell viability by CCK-8 assay. We found that the inhibitory effect enhanced gradually as the concentration of AS1517499 increased whatever in P190 or P210 cells, but AS1517499 had a more significant inhibitory effect on P190 cells than on P210 cells under the same concentration (Fig. 3a). Inhibition of p-STAT6 also restrained the growth of P190 and P210 cells, and the inhibitory effect was more effective in P190 cells (Fig. 3b). The cell cycle distribution was analyzed by FCM. The results demonstrated that inhibition of p-STAT6 resulted in G2/M phase arrest and reduced the number of cells in the S phase in BP190 and SUP-B15 cells (Fig. 3c). In contrast, the G2/M phase arrest was not so remarkable in BP210 and K562 cells under the same treatment of AS1517499 (Fig. 3c). The number of colonies drastically reduced and the size of colonies shrank when p-STAT6 was inhibited in BP190 and SUP-B15 cells (Fig. 3d and e). These results illustrated that the proliferation of P190 cells was significantly suppressed by the inhibition of p-STAT6, and the proliferation inhibition was more obvious in P190 cells than in P210 cells.

### ***Inhibition of p-STAT6 promotes the apoptosis and differentiation in ***Ph+ ***ALL cells***

**Fig. 4:**
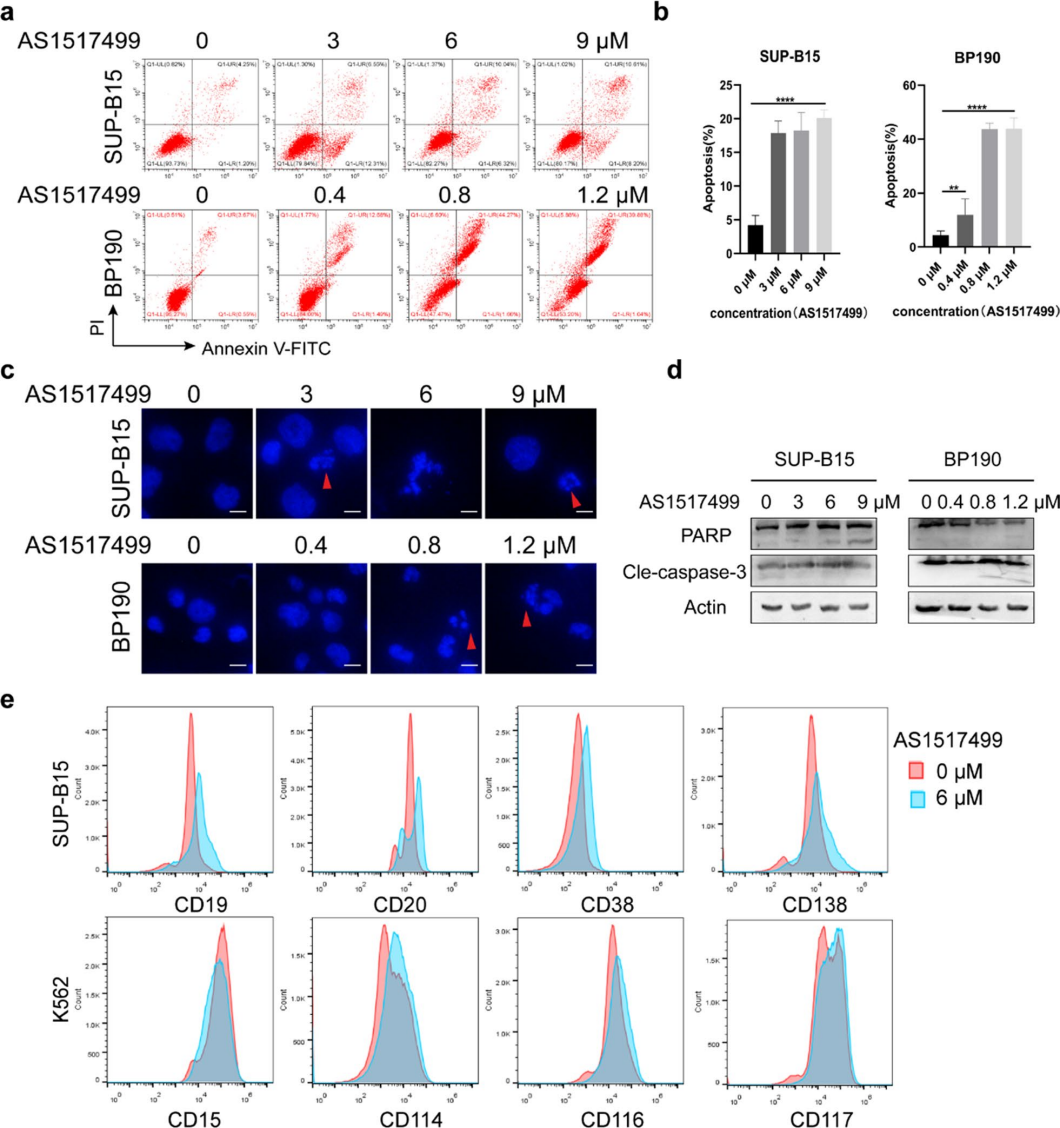
Inhibition of p-STAT6 promotes the apoptosis and differentiation in Ph+ ALL cells. After treatment with different concentrations of AS1517499 for 48 h, **a** cell apoptosis was measured by FCM, and **b** the cell apoptosis rate was analyzed; data are shown as mean ± SD(n = 3); **c** apoptotic morphology was assessed by DAPI staining, and the typical apoptotic cells were indicated by red arrows, scale bar, 10 μM; **d** the expression of PARP and Cle-caspase-3 was measured by western blot assay; **e** the expression of CD19, CD20, CD38, and CD138 in SUP-B15 cells and CD15, CD114, CD116, CD117 in K562 cells were detected by FCM. **p < 0.01, ****p < 0.0001. ns indicates no significant differences

Next, we measured the effect of p-STAT6 inhibition on the apoptosis in P190 cells. We found that the percentage of apoptotic cells increased with the inhibition of p-STAT6 detected by FCM (Fig. 4a, b). There were classical apoptotic morphological changes in the nuclei in the AS1517499 group detected by DAPI staining, such as nuclear condensation and nuclear fragmentation. The nuclei were round and the chromatin distributed evenly in the control group (Fig. 4c). In addition, we determined the levels of cleaved PARP and cleaved caspase-3, which were identified as important markers of apoptosis. The results showed that the cleaved PARP and cleaved caspase-3 were detected when p-STAT6 was inhibited by AS1517499 in BP190 and SUP-B15 cells (Fig. 4d). The results of apoptosis related assays suggested that inhibition of p-STAT6 reduced the ability of P190 cells to resist apoptosis.

The most significant distinction between the two kinds of leukemia caused by BCR-ABL is that CML is related to excessive proliferation of mature myeloid cells, whereas Ph+ B-ALL is associated with blocked B lymphocyte differentiation [2, 12]. Therefore, we explored the effect of p-STAT6 inhibition on leukemic cell differentiation by FCM assay. The results implied that CD19, CD20, CD38 and CD138, the B cell differentiation markers [29, 30], marginally increased in SUP-B15 cells. However, CD15, CD114, CD116, and CD117 associated with myeloid differentiation did not significantly change in K562 cells [31, 32] (Fig. 4e). In conclusion, these data showed that inhibition of STAT6 activation increased the apoptosis in Ph+ ALL cells. And inhibition of p-STAT6 also promoted the differentiation of Ph+ ALL cells but had little effect on Ph+ CML cells.

### *Inhibition of p-STAT6 suppresses the leukemogenesis of P190 but not P210 *in vivo

**Fig. 5:**
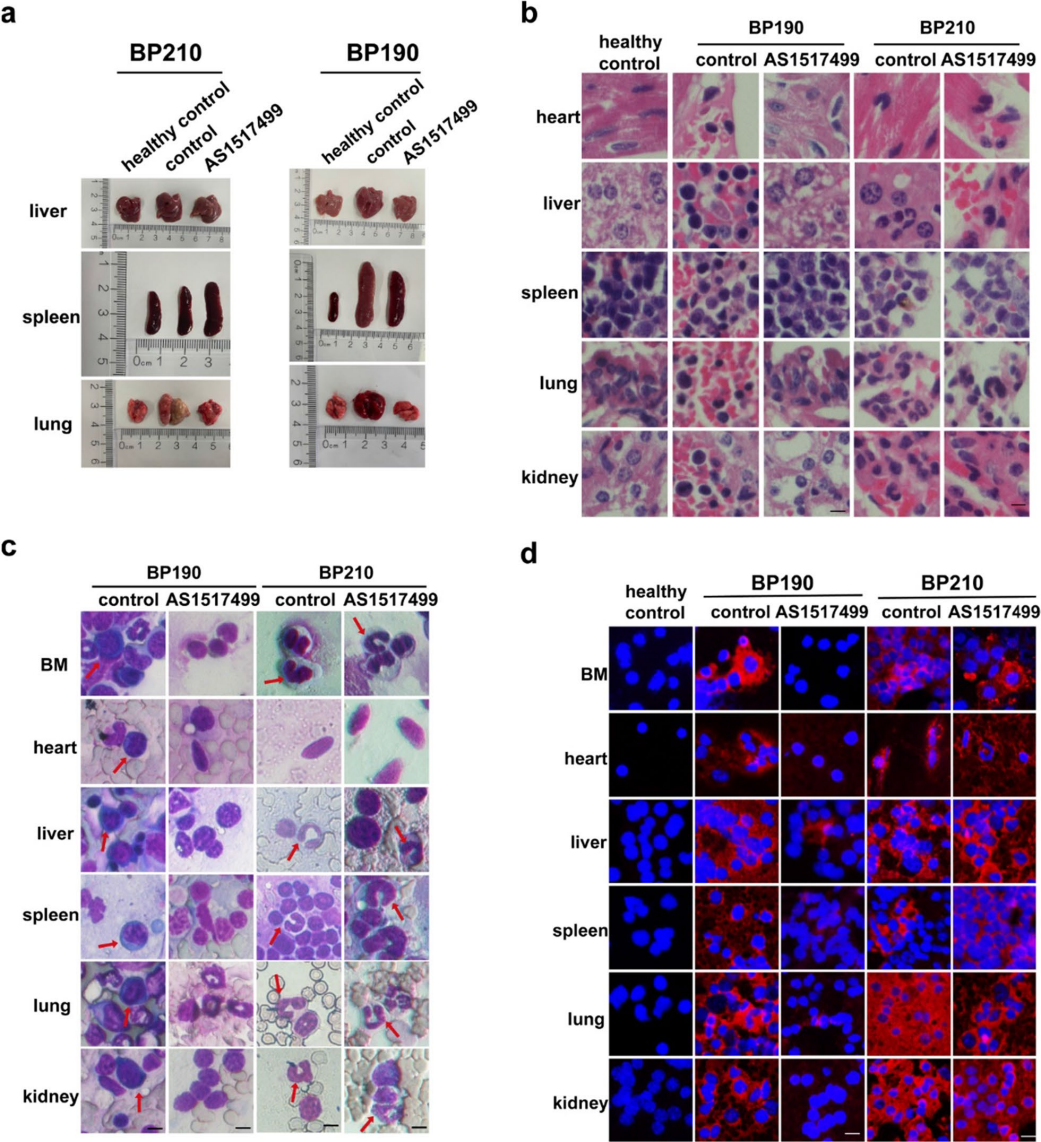
Inhibition of p-STAT6 suppresses the leukemogenesis of P190 but not P210 in vivo. **a** Appearance of liver, spleen, and lung of diseased mice. **b** Leukemic infiltration in the heart, liver, spleen, lung, and kidney was analyzed by HE staining, scale bar, 5 μm. **c** Leukemic infiltration in the heart, liver, spleen, lung, kidney, and BM was analyzed by Wright’s staining, the stab or segmented granulocytes in P210 group and immature lymphocytes in P190 group were indicated by red arrows, scale bar, 5 μm. **d** The expression of BCR-ABL was measured by IF assay, scale bar, 10 μm. Data are presented as the means ± SD. *p < 0.05, **p < 0.01, ***p < 0.001, ****p < 0.0001. ns indicates no significant differences

We confirmed that inhibition of p-STAT6 had a better anti-leukemia effect in vitro in P190 cells than in P210 cells. Furthermore, we wanted to verify the effect of p-STAT6 inhibition in vivo. BP210 or BP190 cells were injected into BALB/C mice by the tail vein to construct Ph+ CML or Ph+ ALL-like mice model respectively. One week later, AS1517499 was injected into mice by intraperitoneal injection with the dose of 20 mg/kg of every other day for five times totally. For the control group, the same volume of PBS was injected (Additional file 1: Fig. S1a). The condition of the mice was monitored every day. The body weight and peripheral white blood cells were measured weekly. The mice were killed when they were in prominent disease states, such as fluffy hair, listlessness, back arching, and chest swelling. Then the critical internal organs, such as heart, liver, spleen, lung, and kidney, were removed for subsequent experiments. The results demonstrated that mice in the AS1517499 group displayed mild symptoms, while mice in the control group showed more severe symptoms in the P190 mouse model, such as obvious hepatosplenomegaly, significant edema and bleeding in the lungs (Fig. 5a). The weight of liver, spleen, or lung was lower in the AS1517499 group compared with the control group (Fig. 1c–e). There was no significant difference in the weight of heart and kidney (Additional file 1: Fig. S1c, d). There was no appreciable variation in the morphology or weight of the vital organs between the AS1517499 group and control group in the P210 models (Fig. 1c–e and Additional file 1: Fig. S1c–e). The WBCs were significantly lower in the treatment group in the P190 model, while there was no difference in WBC counts in the treatment group compared with control group in the P210 model (Fig. 1f). HE and Wright’s staining were performed to detect the infiltration of leukemic cells in tissues. The results of HE staining indicated that the number of immature B lymphocytes infiltrated in the BM, heart, liver, spleen, lung, and kidney was significantly reduced by the treatment of AS1517499 in the P190 model, while AS1517499 did not significantly alleviate the infiltration of leukemic cells in the P210 model (Fig. 5b). The results of Wright's staining were consistent with the results of HE staining (Fig. 5c). Additionally, we detected the expression of BCR-ABL to reflect the infiltration of Ph+ cells in each organ by IF assay. The results showed that the expression of BCR-ABL in the heart, liver, spleen, lung, kidney, and BM was much lower in the AS1517499 group than that in the control group in the P190 model, while this phenomenon was not obvious in the P210 model (Fig. 5d). Finally, AS1517499 significantly prolonged the survival of mice in the P190 model but not in the P210 model demonstrated by the Kaplan–Meier survival curve (Fig. 1j). Taken together, we verified that inhibition of p-STAT6 had a more effective anti-leukemia effect in P190 mouse model than in P210 mouse model.

### ***p-STAT6 promotes the expression of c-Myc as its transcription factor and then accelerates the proliferation of ***Ph+ ***ALL cells***

**Fig. 6:**
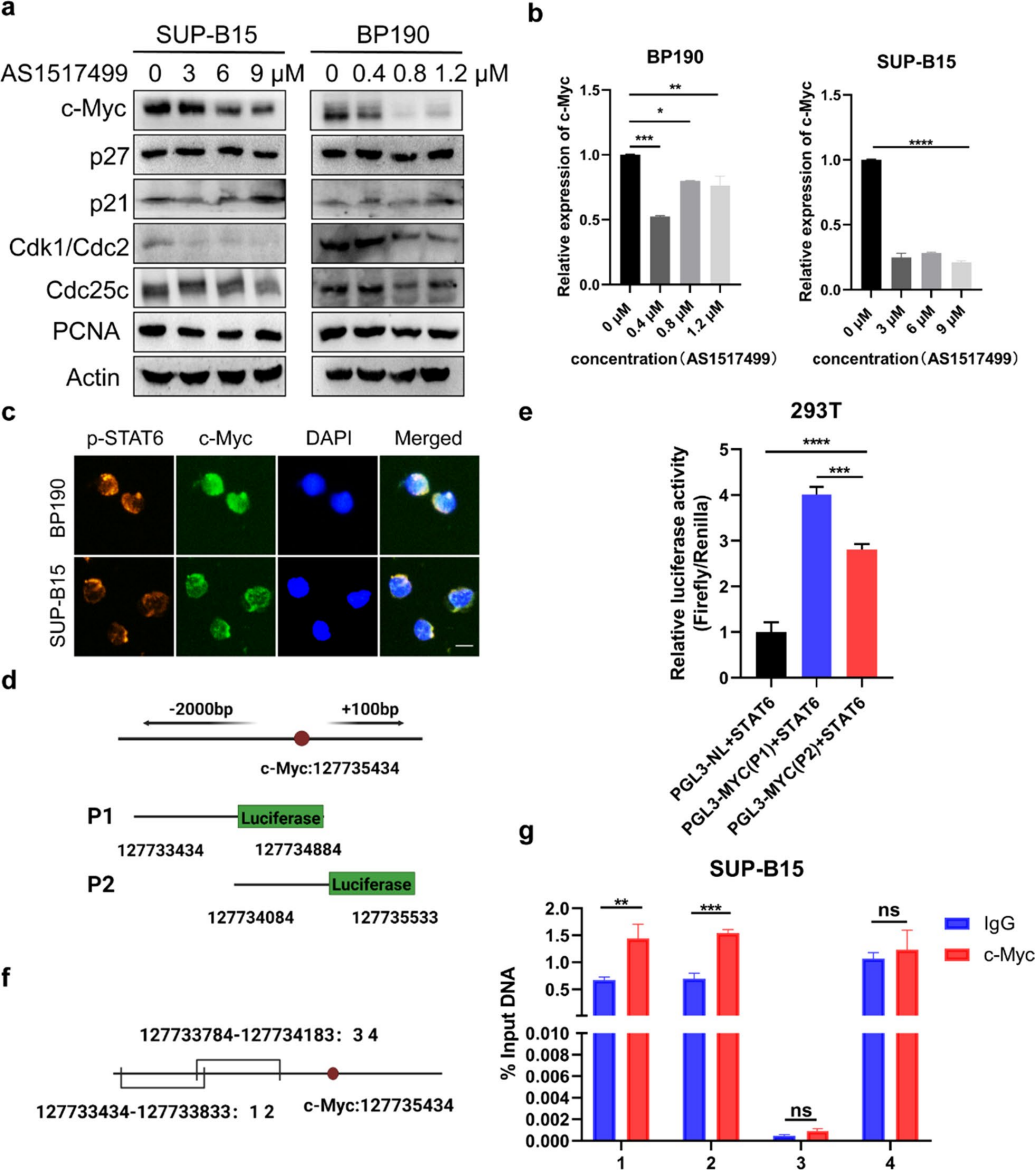
p-STAT6 promotes the expression of c-Myc as its transcription factor and then accelerates the proliferation of Ph+ ALL cells. After treatment with different concentrations of AS1517499 for 48 h, **a** the expression of c-Myc, p27, p21, Cdk1, Cdc25c, and PCNA in BP190 and SUP-B15 cells was measured by western blot assay; **b** the mRNA of c-Myc in BP190 and SUP-B15 cells was measured by qRT-PCR assay. **c** Co-localization of p-STAT6 and c-Myc was detected by IF assay, scale bar, 10 μm. **d** Schematic diagram of the c-Myc promoter region and P1 or P2 gene fragments. **e** PGL3-NL, P1 or P2, and STAT6 plasmids were co-transfected into 293 T cells for 48 h, the luciferase activity was detected by the dual-luciferase assay. **f** Schematic diagram of primers binding to the sequence of c-Myc promoter region. **g** The enrichment of c-Myc gene fragments was analyzed by ChIP-qPCR assay. *p < 0.05, **p < 0.01, ***p < 0.001, ****p < 0.0001. ns indicates no significant differences

We proved that inhibition of STAT6 activation substantially suppressed the proliferation in P190 cell lines but had little effect on P210 cells. That implied p-STAT6 contributed to the progression of Ph+ ALL by regulating the malignant proliferation. Therefore, we focused on the changes of proliferation-related that the expression of Cdk1 and cdc25c declined, while p21 increased and p27 remained unchanged (Fig. 6a), Cdk1 and Cdc25c are both crucial for the G2/M growing stage, while p21 and p27 are Cdk inhibitors [33, 34]. In addition, proliferation-related marker PCNA also decreased (Fig. 6a). These results further proved that the proliferation of Ph+ ALL cells were suppressed by p-STAT6 inhibition. Meanwhile, we found c-Myc was drastically reduced in BP190 and SUP-B15 cells after the treatment of AS1517499 (Fig. 6a). Consistently, the mRNA level of c-Myc also decreased after the treatment of AS1517499 demonstrated by the qRT-PCR assay (Fig. 6b). It is reported that activated STATs family proteins are known to regulate the target gene’s transcription and act as transcription factors [35]. More importantly, c-Myc can restrain the expression of p21 [36, 37].We wondered if p-STAT6 regulated the expression of c-Myc as a transcription factor. We detected the location of p-STAT6 and c-Myc by IF assay. The result showed that p-STAT6 and c-Myc co-located in the nucleus in BP190 and SUP-B15 cells (Fig. 6c). We further validated whether p-STAT6 directly bound to the promoter region of c-Myc by dual luciferase assay and ChIP assay. We divided the potential promoter region of c-Myc into two overlapping segments, which named P1 and P2 respectively, then cloned them into luciferase reporter plasmids (Fig. 6d). 293 T cells were co-transfected by STAT6 plasmid with P1 or P2 plasmid for dual luciferase assay. The results showed that STAT6 enhanced both the activity of P1 and P2, though it was stranger for P1 (Fig. 6e). These results illustrated that STAT6 could bind to the promoter region of c-Myc, especially the region where P1 located. The results of the ChIP assay showed that DNA fragments were strongly enriched in the 1 and 2 primers targeted for the c-Myc promoter sequence arranging from 127,733,434 to 127,733,833, which demonstrated that STAT6 might directly bind to the first 800 bp of the c-Myc promoter region (Fig. 6f, g). Collectively, these results illustrated that p-STAT6 is a transcription factor of c-Myc. Inhibition of p-STAT6 reduced the expression of c-Myc at the transcription level, thereby suppressed the proliferation of Ph+ ALL cells.

### STAT6 is activated by Jak2 not through the classical cytokine pathway

**Fig. 7:**
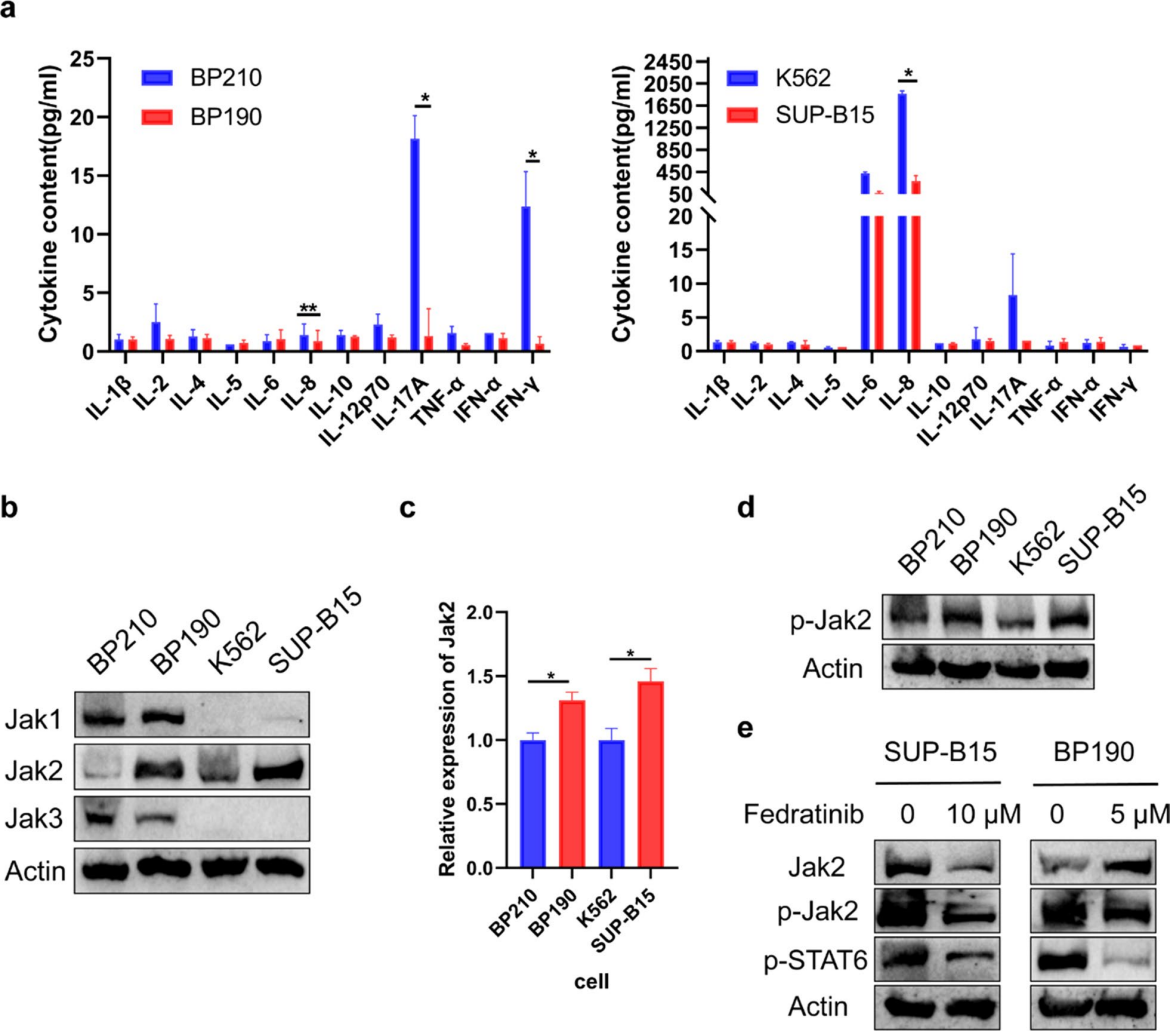
STAT6 is activated by Jak2 not through the classical cytokine pathway. **a** Concentration of cytokines in the supernatant of BP210, BP190, K562, and SUP-B15 cell culture medium were detected by FCM. In BP210, BP190, K562, and SUP-B15 cells, **b** the expression of Jak1, Jak2, and Jak3 was measured by western blot assay; **c** the mRNA of Jak2 was measured by qRT-PCR assay; **d** the expression of p-Jak2 was measured by western blot assay. **e** The expression of Jak2, p-Jak2, and p-STAT6 was measured by western blot assay after treatment with Jak2 inhibitor Fedratinib for 48 h. *p < 0.05

We proved that p-STAT6 was specially elevated in P190 cells but not in P210 cells. However, the reason for the differential activation of STAT6 remains to be investigated. It is reported that STATs are classically activated by cytokines, especially the interleukin (IL) family [38], so we wonder if there are some differences in the cell culture supernatant, including IL-1β, IL-2, IL-4, IL-5, IL-6, IL-8, IL-10, IL-12p70, IL-17A, TNF-α, INF-α, IFN-γ. The results showed that IL-17A and IFN-γ significantly elevated in BP210 cells, but not in K562 cells when compared with BP190 or SUP-B15 cells respectively. IL-8 significantly increased in K562 cells but not in BP210 cells when compared with SUP-B15 or BP190 cells respectively. Except for these three cytokines, there were no significant difference in other nine cytokines between P190 and P210 cells (Fig. 7a). These results demonstrated that the elevated p-STAT6 in P190 cells was not activated by classical cytokine pathway. STATs are also activated by the Jak kinase family [39]. Hence, we investigated the expression of Jaks in Ph+ cells. Our results demonstrated that Jak2 was significantly higher in BP190 and SUP-B15 cells compared with BP210 and K562 cells in the protein and mRNA level (Fig. 7b, c). Then we detected the level of p-Jak2 and found that p-Jak2 was also elevated in P190 cells (Fig. 7d). To further determine whether Jak2 is responsible for the activation of STAT6, we inhibited p-Jak2 with its inhibitor Fedratinib in P190 cells, then tested the level of p-STAT6. We observed that p-STAT6 reduced when p-Jak2 was inhibited (Fig. 7e). That meant p-Jak2 contributed to the activation of STAT6. Taken together, these results illustrated that the higher expression of Jak2 and p-Jak2 but not the cytokine pathway was responsible for the activation of STAT6 in P190 cells.

### ***Inhibition of p-STAT6 improves the sensitivity of ***Ph+ ***ALL cells to IM***

**Fig. 8:**
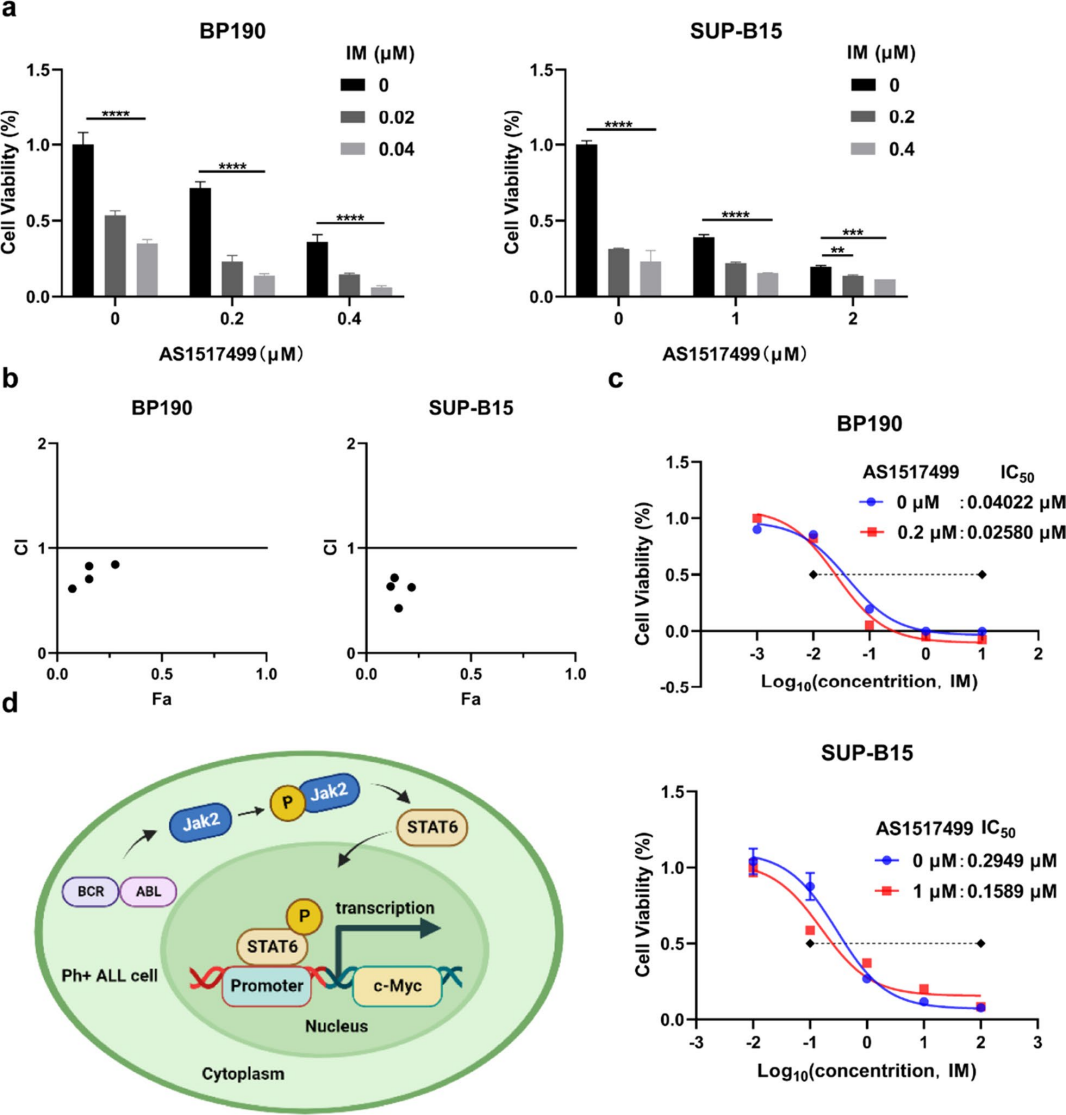
Inhibition of p-STAT6 improves the sensitivity of Ph+ ALL cells to IM. **a** The combined effect of AS1517499 and IM on the viability of BP190 and SUP-B15 cells was evaluated by CCK-8 assay. **b** The combination index of AS1517499 and IM was determined by CompuSyn software in BP190 and SUP-B15 cells. **c** The IC_50_ values of IM combined with a particular concentration of AS1517499 in BP190 and SUP-B15 cells were analyzed by CCK-8 assay. **d** Schematic diagram of the function and mechanisms for inhibiting STAT6 activation in Ph+ ALL cells. In Ph+ ALL induced by P190^BCR-ABL^, the Jak2/STAT6 pathway is activated. Inhibition of STAT6 activation promotes cell apoptosis and suppresses cell proliferation by inhibiting c-Myc transcription. **p < 0.01, ***p < 0.001, ****p < 0.0001

Although it has made great progression with the application of TKIs in the therapy of Ph+ CML, the therapeutic efficiency of TKIs on Ph+ ALL is needed to improve. Concerned that inhibition of p-STAT6 had a powerful anti-leukemia effect, we wondered whether p-STAT6 suppression could improve the sensitivity of Ph+ ALL cells to IM. We treated Ph+ ALL cells with various concentrations of AS1517499 and IM together for 48 h. The result of CCK-8 assay revealed that the combination treatment had a more crucial effect on cell viability than IM or AS1517499 alone whereas in BP190 or SUP-B15 cells (Fig. 8a). Then we calculated the CI values by the CompuSyn software and found all the CI values were less than 1. That meant the combined effect was synergistic effect between IM and AS1517499(CI < 1, = 1, and > 1 represent synergistic, additive, and antagonistic effects, respectively) (Fig. 8b). To further investigated whether the sensitivity of Ph+ ALL cells to IM was enhanced after p-STAT6 inhibition, we detected the IC_50_ value of BP190 or SUP-B15 cells to IM under the condition of p-STAT6 inhibited by 0.2 μM or 1 μM AS1517499. The result of CCK-8 assay showed that the IC_50_ value of IM decreased from 0.04022 μM to 0.02580 μM in BP190 cells and from 0.2949 μM to 0.1589 μM in SUP-B15 cells when p-STAT6 was inhibited by AS1517499 (Fig. 8c). These results suggested that inhibition of p-STAT6 improved the sensitivity of Ph+ ALL cells to IM. In summary, the combination of AS1517499 and IM produced synergistic action and inhibition of p-STAT6 could improve the sensitivity of Ph+ ALL cells to IM. Our findings imply that associative inhibition of BCR-ABL and p-STAT6 may provide a new potential therapy for Ph+ ALL.

## Discussion

The two major BCR-ABL fusion proteins, P210 and P190, which encoded by Ph chromosome, are mainly responsible for Ph+ CML and Ph+ Ph+ ALL respectively [1]. The application of TKIs in Ph + CML patients has recently demonstrated good efficacy and significantly improved their prognosis [9, 10]. However, the efficacy of TKIs is unsatisfactory because of the malignant characteristics of rapid onset and poor prognosis in Ph+ ALL patients, and P190 is often accompanied by additional mutations [14, 15]. Therefore, it is urgent to clarify the regulatory mechanism of P190 in the malignant development of Ph+ ALL, so as to find new therapeutic targets and improve the therapeutic efficacy. Several studies have demonstrated that the aberrant activation of STATs is a critical factor in the emergence of leukemia [19–21]. Additionally, the investigation of the downstream signaling molecules of P190 has revealed STAT6 was specifically activated in P190 cell lines [22–24]. Therefore, we speculated that the more severe leukemia phenotype generated by P190 than P210 might be strongly associated with the additional aberrant activation of STAT6. The effective treatment of Ph+ ALL patients may be made possible by the targeted inhibition of STAT6 activity.

In this study, we confirmed that P190 caused more severe leukemia with malignant phenotype than P210 and STAT6 was specifically activated in P190 cell lines. Then, we used AS1517499 to specifically suppress the activation of STAT6 and found that p-STAT6 inhibition could significantly facilitate apoptosis and inhibit the proliferation of Ph+ ALL cells in vivo and in vitro. Conversely, inhibition of p-STAT6 had little effect on the inhibition in Ph+ CML cells. Noteworthy, we found that the combination of AS1517499 improved the sensitivity of Ph+ ALL cells to IM. Furthermore, inhibition of p-STAT6 somewhat enhanced cell differentiation in Ph+ ALL but had no impact on CML cell differentiation. Then we explored the reason for STAT6 activation and its downstream molecules. We discovered that STAT6 was activated by Jak2, then it stimulated the transcription of c-Myc and promoted the malignant progression in Ph+ ALL induced by P190^BCR-ABL^ (Fig. 8d).

Our results indicated that mice in the P190 group generated an ALL-like disease with a shorter incubation and a higher level of malignancy than mice in the P210. We observed the most significant difference between the two mice models is the change of some vital organs, such as the liver, spleen and lung. The results showed that the hepatosplenomegaly and pulmonary hemorrhage of mice in the P190 group were more severe than those in the P210 group. However, splenomegaly is the main clinical feature of CML patients. The reason for this discrepancy with clinical manifestations may be that in our animal studies, mice in the P210 and P190 groups were killed at the same time. But the onset of Ph+ ALL caused by P190 was rapid, and the mice in the P190 group had typical symptoms, while the onset of CML caused by P210 was slow. Mice in the P210 group did not reach the typical onset period at the time of simultaneous death. Nevertheless, nodules in the spleen of the P210 group can be seen in Fig. 1b, indicating the invasion of leukemia cells, and in Fig. 5a, the splenomegaly of the P210 control group was more serious than that of the healthy control group. Then we examined the therapeutic effect of p-STAT6 inhibition in two mice models. We found that the mice in P190 group that received AS1517499 treatment displayed mild symptoms, while mice in the control group showed more severe symptoms, such as obvious hepatosplenomegaly, significant edema and bleeding in the lungs. There was no appreciable variation in the morphology of the vital organs in P210 group. Therefore, we verified that inhibition of p-STAT6 had a more effective anti-leukemia effect in P190 than that in P210 in vivo.

Next, we confirmed that inhibition of p-STAT6 resulted in G2/M phase arrest, accompanied by the increase of p21 protein but no obvious change of p27 in P190 cell lines. The oncogene c-Myc promotes cell cycle progression of tumor cells partly by inhibiting the synthesis of p21 [36, 37]. We found that the transcription and protein levels of c-Myc were reduced when p-STAT6 was inhibited. We confirmed that p-STAT6 could act as a transcription factor for c-Myc by the double luciferase assay and ChIP-qPCR assay. In general, p-STAT6 promotes the transcription of c-Myc, then increases the expression of p21, in turn to induce G2/M phase arrest and inhibit the proliferation of P190 cells.

It is widely considered that the constitutive activation of STATs is associated with interleukins. For example, IL-4 is a key molecule for STAT6 activation [38, 40]. However, even though p-STAT6 was significantly activated in P190 cells than that in P210 cells, we found no difference in IL-4 or other interleukins content in the culture supernatant of P210 and P190 cell lines. It is reported that BCR-ABL activates the Jak/STAT pathway without the assistance of cytokines or other external stimuli in Ph+ cells [41, 42]. Therefore, we investigated the expression of Jaks, and found the expression of Jak2 and p-Jak2, but not Jak1 and Jak3, up-regulated in P190 cells, which was consistent with p-STAT6. When p-Jak2 was inhibited, p-STAT6 was suppressed conformably. Notably, some research has reported that P190 had higher intrinsic tyrosine kinase activity [43, 44], and our results reflected the expression of p-STAT6 was regulated by BCR-ABL kinase activity. These results imply that the elevated tyrosine kinase activity of P190 compared to P210 may produce a more robust activation of Jak2, which in turn activates STAT6.

In conclusion, our study suggested that Ph+ ALL triggered by P190 exhibits stronger Jak2/STAT6 pathway activation than P210-induced Ph+ CML. Activated STAT6 enhances the transcription of c-Myc then promotes the pathogenicity of Ph+ ALL cells. This could be a crucial mechanism for Ph+ ALL cells to proliferate more malignantly and have a worse prognosis than Ph+ CML. Collectively, this research provides new evidence and insights to illustrate the malignant phenotype and treatment prognosis of leukemia caused by different BCR-ABL subtypes. However, to deeply understand and resolve the pathogenic discrepancies across BCR-ABL subtypes, more researches are required to explore the vital differential molecules and mechanisms.

## Conclusions

Our study demonstrated that Ph+ ALL triggered by P190 exhibits stronger Jak2/STAT6 pathway activation than P210-induced CML. Activated STAT6 enhances the transcription of c-Myc then promotes the pathogenicity of Ph+ ALL cells. This may be a crucial mechanism for the malignant proliferation and worse prognosis of Ph+ ALL cells than Ph+ CML. And this research provides new evidence and insights to illustrate the malignant phenotype and treatment prognosis of leukemia caused by different BCR-ABL subtypes.

## Supplementary Information

**Additional file 1**: **Fig. S1**. **a** Treatment schedule of CML and Ph + ALL-like mice models. **b** Appearance of heart and kidney of diseased mice. The weights of (**c**) heart and (**d**) kidney were measured and statistically analyzed. **e** Representative heart and kidneys in each indicated group were photographed for comparison. ns indicates no significant differences.

## Data Availability

The datasets used and/or analysed during the current study are available from the corresponding author on reasonable request.

## Abbreviations

ABL: Abelson tyrosine kinase
BCR: Breakpoint cluster region
BP190: Ba/F3p190
BP210: Ba/F3p210
BM: Bone marrow
CCK-8: Cell counting kit-8
ChIP: Chromatin immunoprecipitation
DH: Dbl-homology
FBS: Fetal bovine serum
FCM: Flow cytometry
HE: Hematoxylin and eosin
IC_50_: 50% Inhibitory concentration
IF: Immunofluorescence
IL: Interleukin
IM: Imatinib
IMDM: Iscove’s modified Dulbecco’s medium
OD: Optical density
PH: Pleckstrin-homology
Ph: Philadelphia
Ph+ CML: Philadelphia-posive chronic myeloid leukemia
Ph+ ALL: Philadelphia-posive acute lymphoblastic leukemia
PI: Propidium iodide
qRT-PCR: Quantitative real-time PCR
TKIs: Tyrosine kinase inhibitors
WBCs: White blood cells
