# Supplementary material for: Jak2/STAT6/c-Myc pathway is vital to the pathogenicity of Philadelphia-positive acute lymphoblastic leukemia caused by P190BCR-ABL
Source: Cell Commun Signal. 2023 Jan 31;21:27. doi: 10.1186/s12964-023-01039-x (PMC9887777; doi:10.1186/s12964-023-01039-x)
Supplement: Supplementary file 2 — Additional file 1: Fig. S1. a Treatment schedule of CML and Ph + ALL-like mice models. b Appearance of heart and kidney of diseased mice. The weights of (c) heart and (d) kidney were measured and statistically analyzed. e Representative heart and kidneys in each indicated group were photographed for comparison. ns indicates no significant differences. [file 12964_2023_1039_MOESM2_ESM.docx]

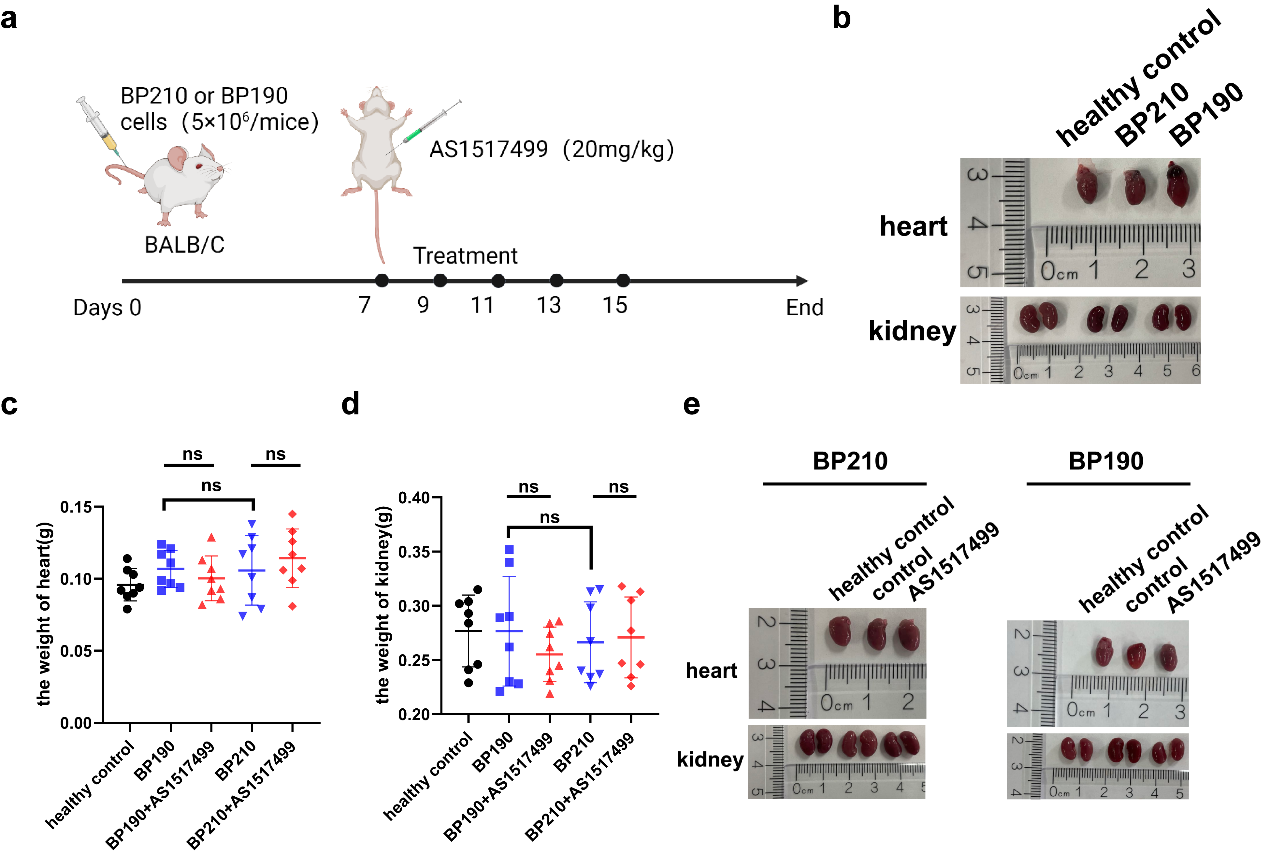


**FigureS1.** **(a)** Treatment schedule of CML and Ph+ ALL-like mice models. **(b)** Appearance of heart and kidney of diseased mice. The weights of **(c)** heart and **(d)** kidney were measured and statistically analyzed. **(e)** Representative heart and kidneys in each indicated group were photographed for comparison. ns indicates no significant differences.
